# Toxicological Safety Evaluation in Acute and 21-Day Studies of Ethanol Extract from Sol*anum lyratum* Thunb

**DOI:** 10.1155/2022/8518324

**Published:** 2022-03-31

**Authors:** XiaoHua Guo, LianJin Weng, LiTao Yi, Di Geng

**Affiliations:** ^1^Instrumental Analysis Center, Huaqiao University, Xiamen 361021, Fujian, China; ^2^Department of Chemical and Pharmaceutical Engineering, College of Chemical Engineering, No. 668, Jimei Road, Huaqiao University, Xiamen 361021, Fujian, China

## Abstract

*Solanum lyratum* (Solanaceae) is a traditional Chinese medicine widely used to remedy cold fever, damp-heat jaundice, herpes, and nephritis dropsy. Despite its obvious therapeutic advantages, few toxicological studies have involved the efficacy and safety of its long-term treatment. To investigate the acute and subchronic toxicity of the extract of 75% ethanol extract of whole *Solanum lyratum* (ESL) after oral administration in mice. In acute toxicity experiment, mice were intragastric administration with ESL at doses of 1000, 2000, 3000, 4000, or 5000 mg/kg for 1 day. In a subchronic toxicity experiment, mice were intragastrically administration with ESL at doses of 180, 360, and 720 mg/kg and 0.9% saline for 21 days. Weight gain, hematological, biochemical, and histopathological analysis of vital organs were evaluated. The presence of aristolochic acid I in ESL was studied using UPLC-QTOF-MS. Phytochemical analysis indicated that the presence of aristolochic acid I in ESL was 0.0025 mg/g. This relatively low concentration is not enough to cause toxicity. In the acute toxicity experiment, neither mortality nor clinical alterations were shown, except for the mild transient diarrhea at 5000 mg/kg. So the LD_50_ value of ESL was assessed to be more than 5000 mg/kg. In the subchronic toxicity experiment, neither mortality nor treatment-related clinical signs were observed. There was a significant increase in body weight, hemoglobin (HB), and urea nitrogen (BUN) after administration with ESL at 180 mg/kg. In addition, the weight of the stomach was increased and the hematocrit (HCT) was decreased after administration with ESL at 360 mg/kg. The changes were not considered treatment-related toxicological effects because the toxicity and histopathological analysis indicate that the extracts are safe for oral administration.

## 1. Introduction

The medicinal plant *Solanum lyratum* Thunb. (Solanaceae) is distributed over the southern areas of the Yangtze River in China. It is also widely used in Korea, Japan, and the Indochina Peninsula. The whole dried *Solanum lyratum* has been used as a traditional Chinese folk medicine for more than 2000 years [1]. *Solanum lyratum* is listed in the “Shen Nong's Herbal Classic” for the treatment of cold fever, damp-heat jaundice, herpes, and nephritis dropsy. Moreover, it is an advantage drug for the treatment of cancer, tumor, rheumatoid arthritis, and leucorrhea [2].

Several pharmacological studies have shown that their phytochemicals have antiangiogenic [1], anticancer [3], antiallergic [4], antibacterial [5, 6], anti-inflammation [7], antioxidant [7, 8], and hepatoprotective effects [9]. Clinically, *Solanum lyratum* was frequently combined with other drugs to treat multifarious cancers, such as lung cancer, liver cancer, gastric cancer, and cervical cancer [10, 11]. In addition, it was applied to diminish inflammation [12]. In recent years, the chemical constituents of *Solanum lyratum* including saponins, flavonoids, organic acids, sesquiterpenoids, steroidal alkaloids, coumarins, and polysaccharides have been isolated [5, 13, 14]. It is reported that herbals containing aristolochic acids may cause toxicity [13]. Alternatively, aristolochic acids I are index ingredients used to determine the content of *Solanum lyratum*.

Despite the *Solanum lyratum* is widely used and has clear therapeutic advantages, there have been few toxicological studies involving its efficacy and safety in a long-term treatment [15], especially considering that subacute toxicity data are demanded to verify these two objectives [16]. Therefore, the present study aims to investigate the toxicological profile of acute and chronic administration of the extract of *Solanum lyratum* (ESL) in mice by focusing on the biochemical and histopathological indexes. The results will provide information for the application of *Solanum lyratum* in clinical therapy.

## 2. Materials and Methods

### 2.1. Plant Materials and Extract Preparation

Whole dried *Solanum lyratum* were purchased from Anxi, Fujian province (Anxi, China). It was identified by Cheng-fu Li, the doctor of Xiamen City Traditional Chinese Medicine Hospital (Xiamen, China). The specimen with voucher number SM 7185 was stored in the herbarium of the department of Chemical and Pharmaceutical Engineering, College of Chemical Engineering, Huaqiao University. 100 g of the *Solanum lyratum* was finely powdered and extracted three times at 25 ± 2°C for 3 h with 75% ethanol. It was filtered and the solvent was partially evaporated under reduced pressure (600 mm Hg) in a rotary evaporator at 60°C. The residual solvent was eliminated in a hot air circulating oven at 45 ± 1°C. The extracts were then lyophilized and kept in a fridge at 4 ± 1°C. The yield of the extract was 16.55%. ESL was dissolved in saline (0.9%) by ultrasonic heating to obtain the required concentration at the time of use.

### 2.2. UHPLC-QTOF-MS Analysis

ESL was dissolved in methanol and microspore filter (0.22 *μ*m), then 10 *μ*L of the filtrate and difference concentrations (1, 3, 5, 10, 20, and 30 *μ*g/ml) of aristolochic acids I (Purity >98%, Sigma-Aldrich, St. Louis, USA) were analyzed by UHPLC-QTOF-MS. It was carried out using a slight modification of the method [17]. The analysis was conducted using an Agilent 1290–6545 UHPLC-QTOF-MS equipped with an Eclipse Plus C 18 column (2.1 × 50 mm, 1.8 *μ*m, Agilent Technologies, USA). The mobile phase consisted of water (A) and acetonitrile (B) (both containing 0.1% formic acid). The gradient conditions of the two mobile phases were as follows: 0–10 min, 25% B and 75% A; 10–20 min, 60% B and 40% A. The flow rate was set at 0.5 ml/min, the mass spectrometer was equipped with an ESI source, and the measure were under positive mode. The concentrations of aristolochic acids I in ESL were calculated from the calibration curve of the reference standard.

### 2.3. Experimental Animals

Male ICR mice (weighing 22–27 g; 5 weeks old; production lot number 2007000577215) were purchased from Fujian Wushi Animal Center (Fuzhou, China). Mice were housed six per cage (320 × 180 × 160 cm) and raised at a temperature of 22 ± 2°C with a relative humidity of 55 ± 5%. The animals were given a standard rodent drying feed diet and water *ad libitum*. Prior to the experiment, the mice were fed adaptively for one week at the animal house. All procedures were approved by the institutional animal care of Huaqiao University (Xiamen, China; approval number HQU 2015016 for toxicity study) and performed in compliance with the published guidelines of the China Council on Animal Care.

### 2.4. Acute Toxicity Studies

In the study evaluating signs of acute toxicity, mice were divided into 6 groups (*n* = 6/group) randomly and fasted for 8 h before the test. Mice were weighed and gavaged with ESL at doses of 1000, 2000, 3000, 4000, and 5000 mg/kg body weight, respectively. Meanwhile, the mice in the 6th group received saline 0.9%. The mice were observed individually at 5, 10, 15, 30, 60, 120, and 240 min after the administration. Surviving mice were observed continually for a period of 14 days. Any clinical signs of toxicity or mortality were monitored and noted according to established standards.

### 2.5. Subchronic Toxicity Studies

In addition, in a repeated dose 21-day subchronic toxicity study, the high-, the medium-, and the low-doses were determined as 720, 360, and 180 mg/kg body weight. Mice were divided into 4 groups including 3 test groups and 1 control group (*n* = 6/group). ESL was administered based on an individual's every 7 day's body weight. During the experiment, clinical symptoms and mortality were observed daily. The signs and symptoms of behavioral alterations were recorded daily, including skin, eyes, gastrointestinal, and peripheral alterations. On the 21st day, mice were overnight fasted and weighed. The blood was sampled from eyes and collected for hematologic analysis and serum biochemical analysis. Then, the mice were euthanized by cervical dislocation [18]. The brain, liver, spleen, kidneys, lungs, heart, and stomach were removed, rinsed in saline 0.9%, and weighed. Animal body weight was measured weekly. The relative organ weights were calculated based on the organ-to-body weight ratio multiplied by 100 [19]. Moreover, tissue fragments of the liver, kidneys, and heart were preserved in 10% formalin solution for histopathological analysis.

### 2.6. Hematologic Analysis

1 mL of red blood cells (RBCs) was collected into the EDTA tubes. Hematologic analysis was tested using an automated haematology analyzer. Hematological evaluations included white blood cell (WBC) count, red blood cell (RBC) count, platelets (PLT) count, hemoglobin (HB), hematocrit (HCT), mean corpuscular volume (MCV), mean corpuscular hemoglobin (MCH), mean corpuscular hemoglobin concentration (MCHC), neutrophils, and lymphocytes.

### 2.7. Serum and Biochemical Analysis

For biochemical analysis, the blood was centrifuged at 3000 ×g for 10 min at room temperature. Serum samples were aspirated off and stored in tubes at −20°C until various analyses (Automatic Biochemical Analyzer, USA). The following clinical biochemistry parameters were determined: total protein (TP), albumin (ALB), globulin (GLB), total bilirubin (TBIL), alanine transaminase (ALT), aspartate aminotransferase (AST), alkaline phosphatase (ALP), total cholesterol (T-CHO), glucose (GLU), urea nitrogen (BUN), and creatinine (CREA).

### 2.8. Histopathological Analysis

All organs and tissues were macroscopically examined for gross pathology. To judge whether histopathological changes were related to the treatment of mice with different concentrations of ESL, vital organs such as the liver, heart, and kidneys were further treated for histopathology. The organs were dehydrated, paraffinized, and embedded according to standard procedures. Sections of 4 *μ*m were stained with hematoxylin and eosin (H&E) in an automated method. The tissues were examined under a microscope in a random order and blinded to the original group.

### 2.9. Statistical Analysis

The results of parametric tests were expressed as mean ± S.E.M. The differences of ratios of organ weight to body weight at the end of 21 days of treatment, body weight changes, as well as biochemicals were analyzed by the one-way ANOVA followed by Dunnett's *post hoc* test between control and test groups (SPSS Inc., Chicago, USA) 20.0. when applicable. For all tests, the level of statistically significance was set at *p* < 0.05.

## 3. Results

### 3.1. UHPLC-QTOF-MS Analysis

**Figure 1:**
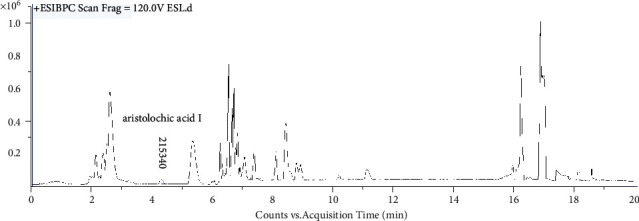
Representative UHPLC-Q/TOF-MS chromatograms of *Solanum lyratum*.

**Figure 2:**
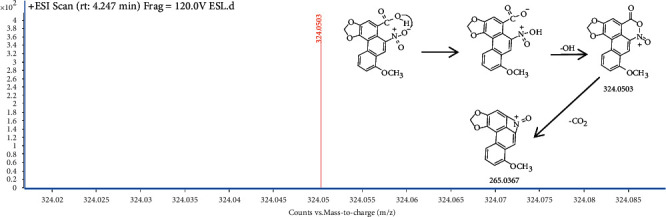
Representative ESI-MS spectra of aristolochic acid I in ESL.

**Figure 3:**
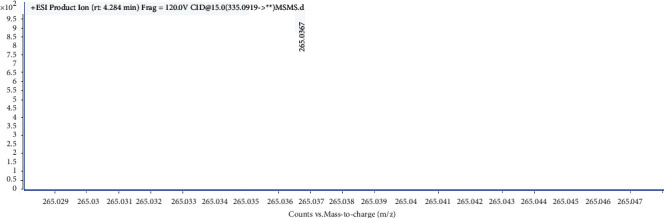
ESI-MS/MS spectra of the fragment ions of aristolochic acid I in ESL.

UHPLC-QTOF-MS analysis revealed the presence of aristolochic acid I in *Solanum lyratum* based on retention times, fragment ions and MS/MS spectra patterns of reference standards [17]. The peaks of aristolochic acid I are visualized at 4.247 min in the *Solanum lyratum* (Figure 1). The ESI-MS spectrum of aristolochic acid I showed an ion at m/z 324.0503 (Figure 2.), corresponding to the [C_17_H_11_NO_7_–OH]^+^. And the generated fragment ions [M–OH–CO_2_]^+^ in MS^2^ (Figure 3). The calibration curve was constructed by plotting the peak areas and the concentrations, which showed good linearity with *R*^2^ = 0.998. The linear equation was *Y* = 8.2012 × 10^4^*X* + 1.031 × 10^4^ (*μ*g/ml). As a result of the calibration curve, 1 g of the extract of *Solanum lyratum* contains 0.0025 mg of aristolochic acid I.

### 3.2. Acute Toxicity

In the present study, no deaths were observed in all groups during the administration periods. Compared with the control group, the test groups presented no treatment-related changes in clinical signs such as external appearance (changes in the skin, fur, and eyes), behavior, mental state, and daily activities. The mice only observed mild transient diarrhea at 5000 mg/kg. Microscopic examination of vital organs including the liver, kidneys, heart, and spleen did not show any changes. Neither mortality nor clinical alterations occurred, so the LD_50_ value of ESL was assessed to be more than 5000 mg/kg.

### 3.3. Mortality and Clinical Observations in Subchronic Toxicity Experiment

There were no ESL treatment-related mortalities in mice after 21 days of oral administration. None of the animals showed obvious morbidity or clinical abnormalities of toxicity such as external appearance (changes in the skin, fur, and eyes), behavior, mental state, and daily activities.

### 3.4. Body Weights and Body Weights Gain in Subchronic Toxicity Experiment

**Figure 4:**
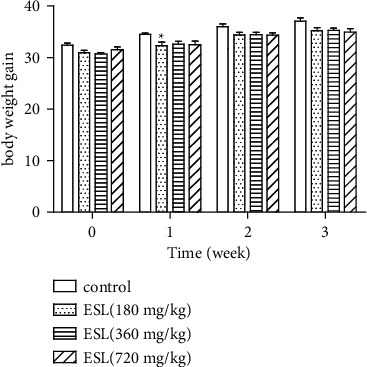
Effect of the ESL, mg/kg i.g., on body weight and accumulated body weight gain in mice. ^*∗*^*p* < 0.05 vs. control group. W0—time zero; W1–W3—every 7 days; the values represent mean ± S.E.M. for 6 animals/group. One way ANOVA, followed by Dunnett's post hoc test.

The effect of the ESL on the body weights and body weights gain are presented in Figure 4. All mice showed a tendency towards increased body weights that were not significantly different from those in the control group. We can only find that mice given 180 mg/kg of ESL induced an increase in body weights compared with the control group at week 1 (*p* < 0.05).

### 3.5. Relative Organ Weights in Subchronic Toxicity Experiment

As shown in Table 1, administration of ESL for 21 days did not produce any abnormal change in the relative organ weights, with the exception of the relative stomach weight (*p* < 0.05) at a dose of 360 mg/kg.

### 3.6. Hematologic Analysis in Subchronic Toxicity Experiment

As shown in Table 2, which reveals haematology parameters, there was a significant decrease in HCT value (*p* < 0.05) at the dose of 360 mg/kg compared with control groups. Meanwhile, compared with the control group, there was a significant increase in HB (*p* < 0.05) at the dose of 180 mg/kg.

### 3.7. Biochemical Analysis in Subchronic Toxicity Experiment

As shown in Table 3, the 21-day treatment with ESL did not lead to abnormalities in serum levels of TP, ALB, GLB, TBIL, ALT, ALP, T-CHO, and CREA. Compared with the control group, there was a significant increase in BUN (*p* < 0.05) at the dose of 180 mg/kg.

### 3.8. Histopathological Examination in Subchronic Toxicity Experiment

**Figure 5:**
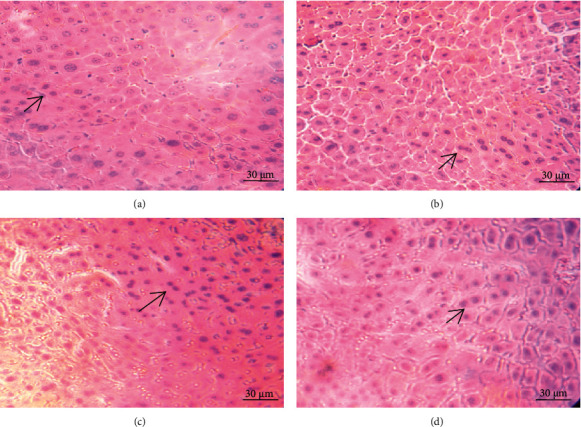
Effects of ESL on hepatic morphological analysis (×40 H&E): control group (a), ESL180 mg/kg group (b), ESL360 mg/kg group (c), and ESL720 mg/kg group (d).

**Figure 6:**
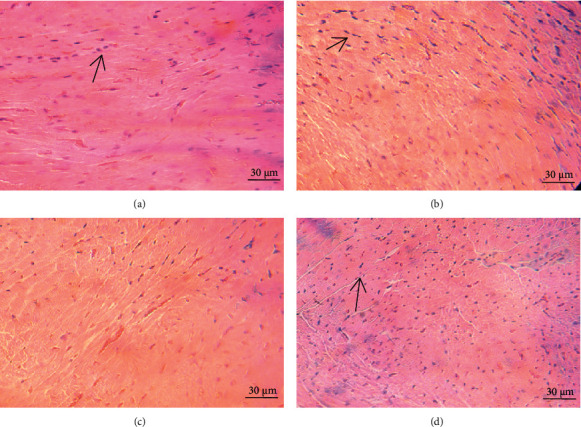
Effects of ESL on heart morphological analysis (×40 H&E): control group (a), ESL180 mg/kg group (b), ESL360 mg/kg group (c), and ESL720 mg/kg group (d).

**Figure 7:**
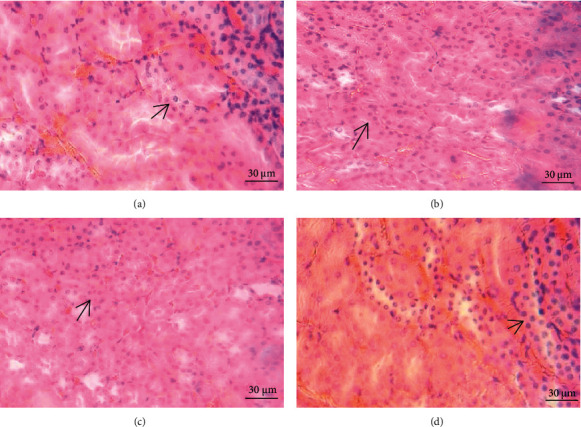
Effects of ESL on renal morphological analysis (×40 H&E): control group (a), ESL180 mg/kg group (b), ESL360 mg/kg group (c), and ESL720 mg/kg group (d).

As depicted in Figures 5–7, microscopic analysis demonstrated that 21 continuous days' administration with 180, 360, and 720 mg/kg ESL had no abnormal changes for architectural and cellular appearances of vital organs (liver, heart, and kidney), compared with the control group.

## 4. Discussion

In recent years, traditional Chinese medicinal herbs have been widely used at home and abroad. People tend to believe natural herbs are safer and have fewer side effects. However, it is necessary to evaluate the toxicity of natural herbs. Research has proved that mice are suitable models for early preclinical safety assessment. Earlier identification of preclinical abnormal symptoms can be predictive of human toxicity and save time, money, and effort spent [20]. Compared with many other medicinal plant species, there are few toxicological studies on the safety of *Solanum lyratum*. The chronic toxicity of ESL is essential for a better understanding of its safety after 21 days of repeated administrations.

Research has reported that aristolochic acids can cause nephrotoxic and cancer. Furthermore, the presence of aristolochic acids is an important marker to identify herbal products containing them [13]. In the current study, the presence of aristolochic acid I in *Solanum lyratum* was 0.0025%. This relatively low concentration is not enough to cause toxicity. But also, *Solanum lyratum* is a traditional Chinese medicinal herb used to treat various cancers in the Asian community hospital.

Although the LD_50_ cannot predict the lethal dose in humans, it provides guidance for dose use in subchronic studies. The present study first investigated the subacute oral toxicity of ESL in mice. The data showed that ESL did not have an obvious toxic effect in mice. In the acute toxicity study, neither mortality nor clinical alterations were shown, except for the mild transient diarrhea at 5000 mg/kg. Therefore, the LD_50_ value of ESL was assessed to be more than 5000 mg/kg [21] and several times higher than 2 mg/kg/d which is the recommended clinical dosage [22]. Therefore, ESL was considered to be nontoxic.

In the subchronic toxicity study, neither treatment-related clinical symptoms nor deaths were recorded during the experiment. During the 21-day subchronic toxicity treatment, a slight weight loss was observed among the mice treated with ESL in the 180 mg/kg group, and it just appeared after 7 days of oral administration. Compared with the control group, the body weight decrease in the 180 mg/kg group was only 6.3%. The amount of weight loss in the mice may be related to a decrease in food intake. Moreover, it was reversible within 21 days of repeated administration [23, 24]. Obviously, the body weight of mice in the 180 mg/kg group increased from the 15th day and gradually returned to the normal level.

Organ weight is an important and sensitive drug indicator to evaluate the toxic effects of test substances in toxicological experiments. Usually, the change of organ coefficient is prior to morphological change [25]. In our study, ESL did not produce any abnormal change in the relative organ weight, with the exception of relative stomach weight in the 360 mg/kg group. This increase was considered to be incidental and not treatment-related since it did not occur in the 720 mg/kg treatment group. The increase of organ coefficients may be due to variations in the sizes of the internal organs and/or body weights of the mice [26, 27]. In addition, by the gastrointestinal mucosa, upon macroscopic and microscopic examinations, the minimal increase of stomach coefficients did not produce abnormal alterations.

WBC, RBC, HB, and PLT are the most sensitive indicators in hematologic analysis, which have a sensitive response to many pathological changes [28]. In addition, hematological examination is a common index to observe the treatment effect and continue treatment or stop treatment. In the study, HCT and HB showed significant changes in hematological parameters, but there was no obvious dose response and the levels of hematological parameters were within the normal range of control values [29].

The liver plays a crucial role in digestion, metabolism, and detoxification of the body. When liver cells are injured by hepatotoxic drugs, the concentrations of serum enzymes such as AST, ALT, and ALP may increase [30]. Alteration was observed in the values of AST, compared with the control group, the enzyme activity of the 360 mg/kg treated group increased by less than 16.7%, and the magnitudes are considered as mild (Table 3). Comprehensive evaluation of the liver enzyme aberration takes into consideration that up to 2.5% of normal person have borderline abnormal enzyme values [31]. In addition, the increase of AST level may also result from extra-hepatic injury, particularly to skeletal muscle [32]. Histopathological evaluations did not show any treatment related damage in the liver. Taken together, the results showed that the increase of AST enzymes was not related to the toxicological effect induced by treatment.

The kidney is a conventional toxic target organ for drug discharge, which can clean metabolic products and poisons. More importantly, it is extremely sensitive to the toxic effects of drugs, and renal damage may lead to an increase of BUN and CREA. The high concentration of serum enzymes such as BUN and CREA is usually considered as one of the sensitive makers of renal damage [33]. Alteration was observed in the values of BUN. The value was greater than the control group at 180 mg/kg (Table 3). However, histopathological evaluations did not show any treatment related changes in the kidney of animals. The value of CREA still remains at a normal level. The change of BUN value was regarded only in one dose and/or was not dose dependent since it did not appear in the 360 and 720 mg/kg treated groups. Taken together, the results showed that the increase of BUN enzymes was not a treatment-related toxicological effect.

Histopathological examination of the heart showed no difference and no apparent abnormality. Microscopic analysis of vital organs indicated that their architectural and cellular appearances were normal and there was no significant difference in the biochemical parameters.

## 5. Conclusion

In conclusion, the present study clearly shows that *Solanum lyratum* has a broad safety margin for clinical therapeutic use. ESL is considered relatively safe in a long-treatment at dose up to 720 mg/kg. Moreover, we did not find any deleterious effect on liver or kidney functions. It is hoped that the result of this experiment will serve as an important reference of ESL for usage as an herbal.

## Figures and Tables

**Table 1:** Effect of the ESL, mg/kg i.g., on relative weights of the internal organs of mice after 21 days of treatment.

Relative organ weights	Control	180 mg/kg	360 mg/kg	720 mg/kg
Brain	1.17 ± 0.03	1.32 ± 0.07	1.21 ± 0.04	1.26 ± 0.02
Liver	4.58 ± 0.12	4.32 ± 0.13	4.43 ± 0.19	4.67 ± 0.31
Spleen	0.29 ± 0.03	0.28 ± 0.02	0.28 ± 0.02	0.30 ± 0.11
Right kidney	0.88 ± 0.03	0.92 ± 0.03	0.81 ± 0.04	0.86 ± 0.04
Left kidney	0.83 ± 0.04	0.88 ± 0.04	0.74 ± 0.05	0.80 ± 0.05
Lung	0.59 ± 0.03	0.70 ± 0.04	0.67 ± 0.03	0.69 ± 0.02
Heart	0.58 ± 0.03	0.63 ± 0.03	0.56 ± 0.03	0.65 ± 0.03
Stomach	1.10 ± 0.08	1.13 ± 0.05	1.34 ± 0.08^*∗*^	1.26 ± 0.04

^*∗*^ *p* < 0.05 vs. control group. Relative organ weights in %; the values represent mean ± S.E.M. for 6 animals/group. One way ANOVA, followed by Dunnett's post hoc test.

**Table 2:** Effect of the ESL, mg/kg i.g., on hematologic parameters in mice.

Haematologic parameters	Control (0 mg/kg)	180 mg/kg	360 mg/kg	720 mg/kg
WBC (10^9/L)	7.67 ± 1.88	7.61 ± 0.93	8.28 ± 2.22	8.09 ± 0.90
RBC (10^12/L)	7.95 ± 0.37	8.22 ± 0.27	7.82 ± 0.29	7.60 ± 0.22
PLT (10^9/L)	858.8 ± 111.55	884.60 ± 81.80	885.5 ± 27.38	842.45 ± 83.09
HB (g/dL)	14.75 ± 0.45	16.83 ± 0.93^*∗*^	14.36 ± 0.34	13.94 ± 0.57
HCT (%)	49.37 ± 2.02	49.79 ± 1.90	45.07 ± 1.30^*∗*^	47.50 ± 1.77
MCV (fL)	62.17 ± 0.74	61.18 ± 2.00	60.22 ± 1.04	61.73 ± 1.59
MCH (pg)	19.05 ± 0.30	19.32 ± 0.50	18.88 ± 0.39	19.30 ± 0.58
MCHC (g/dL)	30.69 ± 0.33	31.09 ± 0.27	31.5 ± 0.51	30.73 ± 0.32
Neutrophils (%)	18.53 ± 1.58	18.94 ± 3.12	20.17 ± 2.02	23.80 ± 3.69
Lymphocytes (%)	74.83 ± 2.51	75.07 ± 1.63	73.85 ± 1.61	69.00 ± 3.10

^*∗*^ *p* < 0.05 vs. control group. The values represent mean ± S.E.M. for 6 animals/group. One way ANOVA, followed by Dunnett's post hoc test.

**Table 3:** Effect of the ESL, mg/kg i.g., on biochemical parameters in mice.

Biochemical parameters	Control	180 mg/kg	360 mg/kg	720 mg/kg
Total protein (g/l)	53.53 ± 2.78	56.68 ± 1.49	57.78 ± 1.92	58.83 ± 1.82
Albumin (g/l)	31.78 ± 2.35	34.08 ± 0.48	35.40 ± 1.10	34.10 ± 0.82
Globulin (g/l)	21.75 ± 0.91	22.60 ± 1.19	22.38 ± 0.83	24.73 ± 2.27
Total bilirubin (umol/l)	1.15 ± 0.95	1.18 ± 0.23	1.20 ± 0.82	1.20 ± 0.58
Alanine transaminase (u/l)	36.25 ± 2.02	34.25 ± 6.61	36.00 ± 10.89	37.25 ± 5.50
Aspertate aminotransferase (u/l)	49.50 ± 5.92	52.75 ± 5.85	57.75 ± 8.07	51.50 ± 9.19
Alkaline phosphatase (u/l)	73.75 ± 4.89	72.50 ± 5.33	74.00 ± 5.42	73.00 ± 10.56
Total cholesterol (mmol/l)	3.41 ± 0.49	3.50 ± 0.38	3.51 ± 0.41	3.36 ± 0.53
Glucose (mmol/l)	5.64 ± 1.52	5.67 ± 0.51	5.55 ± 0.72	5.60 ± 0.62
Urea nitrogen (mmol/l)	5.28 ± 0.57	8.41 ± 0.94^*∗*^	6.13 ± 0.62	5.46 ± 0.35
Creatinine (umol/l)	17.58 ± 4.75	17.85 ± 3.45	17.70 ± 4.57	17.43 ± 3.32

^*∗*^ *p* < 0.05 vs. control group. The values represent mean ± S.E.M. for 6 animals/group. One way ANOVA, followed by Dunnett's post hoc test.

## Data Availability

All the data in relation to this study are included within the manuscript.

## Abbreviations

ESL: Extract of *Solanum lyratum*
ALB: Albumin
HPLC: High-performance liquid chromatographic
GLB: Globulin
WBC: White blood cell
TBIL: Total bilirubin
RBC: Red blood cell
ALT: Alanine transaminase
PLT: Platelets
AST: Aspertate aminotransferase
HB: Hemoglobin
ALP: Alkaline phosphatase
HCT: Hematocrit
T-CHO: Total cholesterol
MCV: Mean corpuscular volume
GLU: Glucose
MCH: Mean corpuscular hemoglobin
BUN: Urea nitrogen
MCHC: Mean corpuscular hemoglobin concentration
CREA: Creatinine
TP: Total protein.
